# Strategies for measuring evolutionary conservation of RNA secondary structures

**DOI:** 10.1186/1471-2105-9-122

**Published:** 2008-02-26

**Authors:** Andreas R Gruber, Stephan H Bernhart, Ivo L Hofacker, Stefan Washietl

**Affiliations:** 1 Institute for Theoretical Chemistry, University of Vienna, Währingerstraße 17, 1090 Wien, Austria; 2 Bioinformatics Group, Department of Computer Science, University of Leipzig, Härtelstrasse 16-18, D-04109 Leipzig, Germany; 3 EMBL-European Bioinformatics Institute, Wellcome Trust Genome Campus, Hinxton, Cambridge CB10 1SD, UK

## Abstract

**Background:**

Evolutionary conservation of RNA secondary structure is a typical feature of many functional non-coding RNAs. Since almost all of the available methods used for prediction and annotation of non-coding RNA genes rely on this evolutionary signature, accurate measures for structural conservation are essential.

**Results:**

We systematically assessed the ability of various measures to detect conserved RNA structures in multiple sequence alignments. We tested three existing and eight novel strategies that are based on metrics of folding energies, metrics of single optimal structure predictions, and metrics of structure ensembles. We find that the folding energy based SCI score used in the RNAz program and a simple base-pair distance metric are by far the most accurate. The use of more complex metrics like for example tree editing does not improve performance. A variant of the SCI performed particularly well on highly conserved alignments and is thus a viable alternative when only little evolutionary information is available. Surprisingly, ensemble based methods that, in principle, could benefit from the additional information contained in sub-optimal structures, perform particularly poorly. As a general trend, we observed that methods that include a consensus structure prediction outperformed equivalent methods that only consider pairwise comparisons.

**Conclusion:**

Structural conservation can be measured accurately with relatively simple and intuitive metrics. They have the potential to form the basis of future RNA gene finders, that face new challenges like finding lineage specific structures or detecting mis-aligned sequences.

## Background

RNA secondary structures serve important functions in many non-coding RNAs and cis-acting regulatory elements of mRNAs [1,2]. They mediate RNA-protein/RNA-RNA interactions in many different biological pathways and some even show enzymatic activity themselves. Functional constraints lead to evolutionary conservation of the RNA structure that in many cases can exceed the level of sequence conservation. Therefore, conserved structures are characteristic evolutionarily signatures of functional RNAs. Most programs developed for the detection of novel functional RNAs rely on these signatures.

QRNA [3] was the first program that detects conserved RNAs. It models RNA structure in a pair of sequences using a stochastic context free grammar. Similarly, EvoFold [4] models the structure of a multiple alignment taking into account a phylogenetic tree (phylo-SCGF). AlifoldZ [5] also analyzes multiple alignments. It uses, however, a thermodynamic folding model based on the RNAalifold algorithm [6]. All three programs fold and evaluate the conservation of the potential RNA at the same time. As a consequence, their scores combine contributions of RNA stability and conservation.

RNAz [7] disentangles both contributions by calculating two separate scores for stability and conservation. The latter, dubbed structure conservation index (SCI), is thus a measure for structural conservation only. Two other programs, MSARi [8] and ddbRNA [9], are available that also calculate a pure conservation score.

In this paper, we revisit the problem and propose a series of other possible strategies to measure structural conservation and compare their performance on a large data set of structural RNA families. The main motivation is to explore alternatives and possible improvements to currently applied measures, especially the SCI used in RNAz. This study seems worthwhile, since comparative approaches like RNAz and others are starting to get extensively used to annotate RNA structures on a genome wide scale [4,10-21]. At the same time, however, the increasing availability of additional sequence data makes it necessary to already reconsider and adapt these strategies. For example, while for the first prototype-screens in the human genome [4,15] only 7 vertebrate genomes were available, we now face the challenge of analyzing alignments of up to 28 species [22]. While the signal from RNA stability is important when only few sequences are available, more emphasis has to be put on the evolutionary signature in future screens. This might improve the specificity of the predictions, a major limitation of current algorithms [23].

However, the results presented here are not only of relevance for comparative *de novo *ncRNA prediction. The SCI, for example, has also been used to measure structural similarity in a clustering approach to find new ncRNA families within one species [13,24]. In principle, conservation measures of that kind could also be useful for general RNA homology search algorithms that combine sequence and structure conservation [25].

Moreover, using a structure conservation measure on an alignment of sequences that are known to have a conserved RNA structure can help to assess the quality of the alignment. This idea has been used to benchmark the performance of multiple alignment programs on structural RNAs [26,27], and more recently to detect mis-aligned sequences and assist in the semi-automatic improvement of RNA alignments [28].

Finally it must be noted that assessing structural conservation, at the same time, means measuring change of RNA structures throughout evolution. Exploring different ways to quantify such structural changes can help inferring structure based phylogenies [29,30] and might improve our understanding of RNA structure evolution [30,31].

## Methods for measuring structural conservation

Structural conservation can be measured on different levels. In the following sections we describe 11 different methods that are based on (i) comparison of predicted minimum free energies (i.e. *not *on their minimum free energy *structures*), (ii) comparison of single structures, (iii) comparison of ensembles of structures representing the whole folding space, and (iv) the two specialized methods used by ddbRNA and MSARi. A short summary of all methods is given in Table 1.

**Table 1:** Overview of methods

Category	Methods	Description	References
Methods based on folding energies	SCI	RNAalifold consensus energy normalized by dividing by the average energy of the single sequences folded independently.	[7]
	SCI_*RN Aeval*_	Evaluation of energies of sequences under the constraint of being forced to fold into the structures of the other sequences in comparison to the unconstrained energies.	this work
Methods based on single structures	Base-pair distance	Number of base-pairs not shared by two structures.	[64]
	Mountain metric	Distance as the difference of two mountain functions, which give the number of base-pairs enclosing a position.	[40]
	Tree editing	Based on the representation of RNA secondary structures as trees. A distance is deined as the cost of transforming one tree into the other.	[41-43]
Methods considering the entire folding space	Ensemble distance	Base-pair distance extended to compare ensembles of structures.	this work
	Ensemble mountain metric	Distance as the difference of two mountain functions, which give the number of base-pairs that are, on average, expected to enclose a position.	[47]
	RNApdist like distance	Distance measure based on the comparison of vectors of probabilities of being paired upstream, paired downstream, and unpaired.	[33,48]
	RNAshapes	Similarity measure based on probabilities of abstract shapes.	[49]
Other Methods	ddbRNA	Evaluates compensatory mutations in all possible stem loops in all sequences of an alignment.	[9]
	MSARi	Evaluation of the statistical significance of short, contiguous potential base-pair regions under different distribution models.	[8]

### Methods based on folding energies

The idea to evaluate structure similarity indirectly through the minimum free energy (MFE) rather than by direct comparison of the structure itself seems to be counter-intuitive at the first glance. The principle, however, becomes clear when considering the RNAalifold algorithm. RNAalifold implements a consensus folding algorithm for a set of aligned RNA sequences. It extends standard dynamic programming algorithms for RNA secondary prediction [32] by averaging the energy contributions over all sequences and incorporating covariation terms into the energy model to reward compensatory mutations and to penalize non-compatible base-pairs. This procedure results in a "consensus MFE" for the alignment. The absolute value of the consensus MFE is of little value to assess the conservation of structures since it mainly reflects the folding energy that is heavily dependent on the nucleotide composition and the length of the alignment. Therefore, the consensus MFE *E*_*cons *_is normalized by the average MFE ${\bar{E}}_{single}$ of the single sequences as computed by RNAfold giving the structure conservation index

(1) $$SCI=E_{cons}/{\bar{E}}_{single}$$

If the sequences show equally stable folding energies if forced to fold into a common structure compared to being folded independently, this indicates a conserved structure and the SCI is high. The lower bound of the SCI is zero, indicating that RNAalifold is not able to find a consensus structure, while a SCI close to one corresponds to perfect structure conservation. Compensatory mutations adding additional bonus energies to the consensus MFE can even give rise to a SCI higher than one.

The SCI, as given above, requires the computation of a consensus structure for the whole alignment. Alternatively, one can consider formulating a similar measure based on pairwise comparisons of all sequences. To this end, the folding energy of each sequence is evaluated when forced to fold into the structures of the other sequences. The pairwise SCI for an alignment A is given by

(2) $$SCI_{RN\;Aeval}(A)=\frac{\sum_{\begin{array}{c} x,y\in A\\ x\neq y \end{array}}E(x|S_{y})}{(N-1)\sum_{x\in A}E(x|S_{x})}$$

where *E*(*x*|*S*_*y*_) denotes the free energy of sequence *x *when adopting the minimum free energy structure *S*_*y *_of sequence *y*, and *N *is the number of sequences in the alignment. The free energies for a given sequence in a given structure can be easily evaluated with the program RNAeval from the *Vienna RNA *package [33]. Therefore, we refer to this method as the "RNAeval" method.

### Methods based on single structures

A more intuitive way to assess structural similarity is by comparing structures themselves rather than comparing the energies associated with these structures. Conservation measures derived from various structure metrics are described in this section. Unlike the energy based methods from the previous section that are inherently linked to thermodynamic folding, the following methods do not depend on the way of how structures are predicted. There are several different ways, like thermodynamic energy minimization [34], kinetic folding [35] or probabilistic models [36-38], but the choice of the method will not influence the underlying concept. However, since the goal of this study is not to compare the accuracy of different folding algorithms, we use here exclusively energy minimization (RNAfold) to ensure comparability between all methods.

#### Base-pair distance

The most simple distance measure between two *sequences *is the Hamming distance, i.e. the number of positions with different nucleotides. For RNA structures, one could think of calculating the Hamming distance of two strings in dot bracket notation with the three characters "(", ".", ")". However, this does not account for the correlations between the opening and closing positions that are characteristic for the structure.

An alternative to the Hamming distance more suitable for secondary structures is the so-called base-pair distance. The base-pair distance between to RNA secondary structures *S*_*x *_and *S*_*y *_is defined as the number of base-pairs not shared by the two structures. Formally it can be described in terms of set theory, where the base-pair distance corresponds to the cardinality of the symmetric set difference:

(3) $$\begin{array}{c} d_{BP}(S_{x},S_{y})=|(S_{x}\backslash S_{y})\cup (S_{y}\backslash S_{x})|=|S_{x}\cup S_{y}|-|S_{x}\cap S_{y}|\\ =|S_{x}|+|S_{y}|-2|S_{x}\cap S_{y}|=\sum_{i<j}\left(\delta_{ij}^{x}+\delta_{ij}^{y}-2\delta_{ij}^{x}\delta_{ij}^{y}\right) \end{array}$$

with $\delta_{ij}^{x}$ = 1 if (*i*,*j*) is a base-pair of structure *S*_*x*_, and $\delta_{ij}^{x}$ = 0 otherwise. *d*_*BP *_itself is not a suitable measure for comparison as long it is not set in relation to the union of the base-pairs in *S*_*x *_and *S*_*y*_. The normalized base-pair distance scaled to the interval [0, 1] between two structures is given by

(4) $$D_{BP}(S_{x},S_{y})=\frac{\left|S_{x}\cup S_{y}|-|S_{x}\cap S_{y}\right|}{\left|S_{x}\cup S_{y}\right|}=\frac{\sum_{i<j}\left(\delta_{ij}^{x}+\delta_{ij}^{y}-2\delta_{ij}^{x}\delta_{ij}^{y}\right)}{\sum_{i<j}\left(\delta_{ij}^{x}+\delta_{ij}^{y}-\delta_{ij}^{x}\delta_{ij}^{y}\right)}$$

The overall score for a multiple alignment A can either be calculated as the average of all pairwise sequence comparisons

(5) $$\frac{2}{(N-1)N}\sum_{\begin{array}{c} x,y\in A\\ x>y \end{array}}D_{BP}(S_{x},S_{y})$$

or as the average of all comparisons of each sequence to a consensus structure

(6) $$\frac{1}{N}\sum_{x\in A}D_{BP}(S_{x},S_{consensus})$$

If not stated otherwise, also all other methods that are based on pairwise comparisons can be calculated either as the average over all (*N *- 1)*N*/2 pairwise comparisons, or the average of all *N *comparisons to the consensus structure.

#### Mountain metric

The mountain metric is based on the mountain representation of RNA secondary structures [39] and follows the idea that the distance between two structures *S*_*x *_and *S*_*y *_can be expressed as the difference of the two mountain graphs. For this purpose, a *l*_*p*_-norm can be defined that induces a metric $d_{M}^{p}$ on two secondary structures *S*_*x *_and *S*_*y *_as the difference of the two mountain functions *m*(*S*_*x*_) and *m*(*S*_*y*_) [40]:

(7) $$d_{M}^{p}(S_{x},S_{y}):=||m(S_{x})-m(S_{y})||:=(\sum_{k=1}^{n}|m_{k}(S_{x})-m_{k}(S_{y}){|}^{p}{)}^{\frac{1}{p}}$$

The mountain function *m*_*k*_(*S*) is defined as the number of base-pairs enclosing position *k*. The effect that base-pairs are weighted differently can be overcome by scaling each base-pair to the range it spans.

(8) $$m_{k}(S)=\sum_{i<k}\sum_{k<j}\frac{1}{j-i-1}$$

As $d_{M}^{p}$ is expected to grow with the length of sequences, we are in the need of defining a normalized distance measure to be able to compare distances for sequence pairs of different length. The maximal distance of a secondary structure *S*_*max *_on a sequence of length *n *to the open chain *S*_*open *_is obtained if *S*_*max *_is a stem of maximal height (⌊(*n *- 3)/2⌋), which is a hairpin loop enclosing three unpaired bases. The normalized mountain metric $D_{M}^{p}$ is then defined as the ratio of the distance $d_{M}^{p}$ (*S*_*x*_, *S*_*y*_) of two secondary structures with length *n *to the maximal distance $d_{M}^{p}$ (*S*_*max*_, *S*_*open*_) at length *n*:

(9) $$D_{M}^{p}(S_{x},S_{y})=\frac{d_{M}^{p}(S_{x},S_{y})}{d_{M}^{p}(S_{max},S_{open})}$$

#### Tree editing

RNA secondary structures can be represented as ordered, rooted trees [41-43]. The tree representation can be deduced from the dot-bracket notation (characters "(" and ")" correspond to the 5' base and the 3' base in the base-pair, respectively, while "." denotes an unpaired base), as the brackets clearly imply parent-child relationships. The ordering among the siblings of a node is imposed by the 5' to 3' nature of the RNA molecule. To avoid formation of an unconnected forest of trees, a virtual root has to be introduced.

The tree representation at full resolution without any loss of information with regard to the dot-bracket notation can be derived by assigning each unpaired base to a leaf node and each base-pair to an internal node. The resulting tree can be rewritten to a *homeomorphically irreducible tree *(HIT) by collapsing all base-pairs in a stem into a single internal node and adjacent unpaired bases into a single leaf node [43]. Each node is then assigned a weight reflecting the number of nodes or leaves that were combined.

Shapiro proposed another encoding that retains only a coarse-grained shape of a secondary structure [41]. This is useful in the case of comparison of major structural elements of a RNA molecule but it comes along with a loss of information (cf. section "Abstract shapes"). A secondary structure can be decomposed into stems (S), hairpin loops (H), interior loops (I), multi-loops (M), and external nucleotides (E). While external nucleotides are assigned to a leaf, unpaired bases in a multi-loop are lost. The weighted coarse-grained approach compensates the effect of information reduction at least by assigning to each node or leaf the number of elements that were condensed to it.

Tree editing induces a metric in the space of trees and hence a metric in the space of RNA secondary structures. An edit script, which is a series of edit operations, namely deletion, insertion and relabeling of a node, each assigned a cost can transform any tree *T*_*x *_into any other tree *T*_*y*_. The distance between two trees *d*(*T*_*x*_, *T*_*y*_) is then defined as the cost of the edit script with minimal cost. Normalization of the tree editing distance is done by comparing the distance of two trees *d*(*T*_*x*_, *T*_*y*_) to the sum of the costs of deleting either of the two secondary structures, where • denotes a tree consisting solely of a root:

(10) $$D_{T}(T_{x},T_{y})=\frac{d(T_{x},T_{y})}{d(T_{x},•)+d(•,T_{y})}$$

Among the methods used here, tree editing is the only one that can act on structures of unequal length. In this work we will focus on two different implementations of tree editing. RNAdistance [33] a tool from the *Vienna RNA *package implements a tree editing algorithm initially proposed by Shapiro [41] and acts on the full representation, HIT representation [43], coarse-grained and weighted coarse-grained representation [41]. Allali & Sagot [44] pointed out some shortcomings of the classic tree editing operations and introduced novel editing operations called *node-fusion *and *edge-fusion*, implemented in the program MiGaL. MiGaL uses a new concept of encoding trees at different levels of abstraction called layers [45], which are interconnected to each other via vertex coloring operations.

### Methods considering the entire folding space

#### Distance of structure ensembles

Because the stabilizing energies of base-pair formation are in the same energy range as the thermal energy, RNA molecules in physiological conditions are far away from being caged into one rigid secondary structure. Instead, one usually observes an ensemble of RNA structures, which can be represented by an energy weighted Boltzmann distribution. McCaskill proposed a dynamic programming algorithm [46] that allows to efficiently compute the partition function *Q*, where Δ*G *is the conformational Gibb's Free Energy change, *R *is the gas constant, *T *is the absolute temperature, and S is the ensemble of possible secondary structures.

(11) $$Q=\sum_{S\in S}e^{\frac{-\Delta G(S)}{RT}}$$

The probability of a single structure *S *is then given by

(12) $$P(S)=\frac{e^{-\Delta G(S)/RT}}{Q}$$

and hence the probability of a single base-pair (*i*, *j*) is

(13) $$p_{ij}=\sum_{S\in S}P(S)\delta_{ij}^{S}$$

where $\delta_{ij}^{S}$ is one if (*i*, *j*) is a base-pair of structure *S*, and zero otherwise. Using these assumptions the equation of the base-pair distance can be remodeled to calculate the average base-pair distance $\langle d_{BP}(S_{y},S_{x})\rangle$ between all structures of the two ensembles $S_{x}$ and $S_{y}$.

(14) $$\begin{array}{lll} \langle d_{BP}(S_{x},S_{y})\rangle & = & \sum_{S_{x}\in S_{x}}\sum_{S_{y}\in S_{y}}\left[P(S_{x})P(S_{y})\sum_{i<j}\left(\delta_{ij}^{x}+\delta_{ij}^{y}-2\delta_{ij}^{x}\delta_{ij}^{y}\right)\right]\\ & = & \sum_{i<j}[\sum_{S_{x}\in S_{x}}P(S_{x})\delta_{ij}^{x}\sum_{S_{y}\in S_{y}}P(S_{y})\\ & & +\sum_{S_{y}\in S_{y}}P(S_{y})\delta_{ij}^{y}\sum_{S_{x}\in S_{x}}P(S_{x})\\ & & -2\sum_{S_{x}\in S_{x}}P(S_{x})\delta_{ij}^{x}\sum_{S_{y}\in S_{y}}P(S_{y})\delta_{ij}^{x}]\\ & = & \sum_{i<j}\left[p_{ij}^{x}+p_{ij}^{y}-2p_{ij}^{x}p_{ij}^{y}\right]=\sum_{i<j}p_{ij}^{x}(1-p_{ij}^{y})+p_{ij}^{y}(1-p_{ij}^{x}) \end{array}$$

As one can see in the last line, this corresponds to the the naïve approach of multiplying the probability of the base-pair (*i*, *j*) in the ensemble $S_{x}$ with the probability of not expecting the base-pair (*i*, *j*) in the ensemble $S_{y}$ and vice versa. Taking a closer look at equation 14, one can see that the distance between the structure ensemble of one sequence ⟨*d*_*BP*_($S_{x}$, $S_{x}$)⟩ is not zero. Instead, it is the average distance between the structures in the ensemble, referred to as ensemble diversity. As we are interested in the distance between two ensembles, one has to subtract the average of the ensemble diversities to ensure identity and symmetry. The *ensemble distance *$D_{ensemble}(S_{x},S_{y})$ between two ensembles $S_{x}$ and $S_{y}$ is then defined as follows:

(15) $$\begin{array}{lll} D_{ensemble}(S_{x},S_{y}) & = & \langle d_{BP}(S_{x},S_{y})\rangle -\frac{1}{2}(\langle d_{BP}(S_{x},S_{x})\rangle +\langle d_{BP}(S_{y},S_{y})\rangle )\\ & = & \sum_{i<j}\left[p_{ij}^{x}+p_{ij}^{y}-2p_{ij}^{x}p_{ij}^{y}\right]-\frac{1}{2}\sum_{i<j}\left[2p_{ij}^{x}-2p_{ij}^{x2}\right]-\frac{1}{2}\sum_{i<j}\left[2p_{ij}^{y}-2p_{ij}^{y2}\right]\\ & = & \sum_{i<j}\left[p_{ij}^{x2}+p_{ij}^{y2}-2p_{ij}^{x}p_{ij}^{y}\right]=\sum_{i<j}{\left(p_{ij}^{x}-p_{ij}^{y}\right)}^{2} \end{array}$$

The result, which is simply the sum over the squared differences of the pair probabilities, is a very intuitive distance measure of two ensembles. Note that this measure is not a metric since the triangle equation is not fulfilled. However, $\sqrt{D_{ensemble}(S_{x},S_{y})}$ is a metric, as it corresponds to the euclidean distance between two vectors.

Also the mountain metric approach previously discussed can be readily extended to incorporate base-pairing probabilities [47]. The mountain function *m*_*k*_(*S*) gives then the number of base-pairs that are expected to enclose position *k *on average:

(16) $$m_{k}(S)=\sum_{i<k}\sum_{k<j}\frac{p_{ij}}{j-i-1}$$

#### Distance of one dimensional pair-probability vectors

Another method to compare the folding space of two RNA sequences is by aligning one dimensional base-pairing probability vectors [48], as implemented in the program RNApdist. From all base-pairing probabilities of base *i *the probabilities of being paired downstream ($p_{i}^{<}$), paired upstream ($p_{i}^{>}$), and unpaired ($p_{i}^{o}$) are computed:

(17) $$\begin{array}{ccc} p_{i}^{<}=\sum_{i,j}p_{ij} & p_{i}^{>}=\sum_{i>j}p_{ij} & p_{i}^{o}=1-p_{i}^{<}-p_{i}^{>} \end{array}$$

In this study we use a RNApdist-like variant *D*_*RN Apdist *_as a distance measure for a precomputed alignment of two sequences *x *and *y *as follows:

(18) $$D_{RN\;Apdist}(x,y)=\frac{1}{L}\sum_{i}^{L}\delta_{i}(x,y)$$

where *L *is the length of the alignment and *δ *given by

(19) $$\delta (x,y)=\{\begin{array}{ll} 1-\sqrt{p_{i}^{<}(x)p_{i}^{<}(y)}-\sqrt{p_{i}^{>}(x)p_{i}^{>}(y)}-\sqrt{p_{i}^{o}(x)p_{i}^{o}(y)} & \text{aligned position}\\ 0 & \text{inserted or deleted position} \end{array}$$

#### Abstract shapes

Giegerich *et al*. [49] introduced the concept of *abstract shapes*, coarse-grained abstractions of full secondary structures. The current implementation of RNAShapes offers five levels of abstraction and partitions the folding space into structural families represented by the different shapes. The probabilities for shapes are calculated by summing up the probabilities of all structures that are assigned to the same shape [50,51].

A pairwise similarity measure s comparing two shape spaces $S_{x}$ and $S_{y}$ can be defined as follows, where *p*(*S*|*x*) and *p*(*S*|*y*) is the probability of shape *S *given sequence *x *and *y*, respectively.

(20) $$s(x,y)=\sum_{S\in S_{x}\cup S_{y}}\sqrt{p(S|x)p(S|y)}$$

### Other Methods

One key characteristic of conserved structures are compensatory mutations. Compensatory mutations that maintain the secondary structure will accumulate as this helps keeping the RNA molecule functioning. While all methods described so far include structure predictions and only indirectly depend on such compensatory mutations, Di Bernardo *et al*. [9] proposed a method that is solely based on the existence of compensatory mutations. ddbRNAcounts compensatory mutations in all possible stem loops in all sequences of an alignment without making use of a folding model of any sort. In this paper we will use the number of compensatory mutations per length that is calculated by ddbRNA as measure for structural conservation.

Coventry *et al*. [8] follow with their MSARi algorithm a similar but more elaborate strategy than that of ddbRNA. Decision about structural conservation is made upon statistical significance of short, contiguous potential base-paired regions. The partition function implementation of RNAfold is used to predict base-pair probabilities. Each base-pair (*i*, *j*) with a base-pairing probability higher than 5% is then examined individually. For each sequence in the alignment a window of length seven is centered on nucleotide *i *and compared with a series of windows centered around *j *± {0, 1, 2} (to compensate slight mis-alignments). The window pair with the maximal number of reverse complementary positions is chosen for further analysis, which is the evaluation of the probability of seeing at least as many compensatory positions against a null-hypothesis distribution for random mutations. The estimation of the significance of observed base-pairs is then used to assess the total significance of the alignment.

The main interest of this paper is to detect structural similarities in a given alignment. Clearly, the problem of calculating the alignment and detecting a conserved structure is closely related. For example, structural alignment algorithms based on the Sankoff algorithm [52] can be used to detect conserved structures [18,19] or homologues of a given structure [53]. Aligning sequences requires a notion of sequence similarity and, therefore, sequence substitution models of RNAs have been developed. Examples are the RIBOSUM matrices for the homology search program RSEARCH [53] or a specifically parametrized general time reversible (GTR) model for ITS2 sequences [54]. We do not cover methods here that are primarily focused on the alignment problem, such as Sankoff based algorithms, nor methods that combine sequence and structure comparison such as the family of edit distances on arc annotated sequences by Zhang and coworkers [55] (although RNAdistance represents a special case of these) or tree alignment as implemented in RNAforester [56]. If used with a sequence weight of zero, we would expect these methods to give similar results to the RNAdistance tree editing. Liu & Wang [57] recently proposed a method for RNA secondary structure similarity analysis based on the Lempel-Ziv compression algorithm. However, since the authors do not provide an implementation of their method it could not be considered in this study.

### Benchmarking

To assess the performance of the various methods to detect conserved RNA structures in multiple sequence alignments, we conducted a comprehensive benchmark on the *BRAliBase *database version 2.1 [27]. This database provides a reasonable sized data set of homologous RNAs of different families. In addition to the structural alignments provided by the database we generated for each alignment a corresponding sequence-based alignment using CLUSTAL W [58].

Despite their shortcomings, pure sequence based alignments represent a more realistic scenario because structural alignments are not always available in real life situations (e.g. genome wide screens). There are many structural alignment programs available. As mentioned before, the problem of structural alignment and finding structural similarities is closely related. However, we do not want to compare the efficiency of different alignment programs and thus stick with the two extreme cases of purely sequence based alignments and manually curated reference alignments. At this point we want to mention, that our results might be interesting for some of the alignment algorithms. For example, the heuristic algorithm of CMFinder [59] uses a distance measure based on tree editing in one of the first alignment steps.

As a negative control of alignments that do not harbor a conserved structure we randomized each alignment of the database by shuffling. The procedure is described in detail in reference [5]. It is as conservative as possible and keeps the most relevant alignment parameters like base composition, conservation patterns, gap-patterns etc. intact while any correlation arising from the original structure is efficiently removed.

The sensitivity to detect a conserved RNA structure depends on the sequence variation in the alignment. It is difficult to detect any signature of a conserved structure in alignments with high sequence identity. The more sequence changes in the alignment the more information is available. The overall "information content" is thus dependent on (i) the divergence of the sequences and (ii) the number of the sequences in the alignment. A common measure describing sequence variation in a multiple sequence alignment is the average pairwise sequence identity (API). Although this measure is widely used, it is only capable of assessing sequence variation, and does not take the number of sequences of the alignment into account. We found it helpful to use a combined measure for the content of evolutionary information for presenting the results of our analysis. We used the normalized Shannon entropy *H*. In the case of alignments of RNA sequences we are dealing with an alphabet Σ = {A,C,G,U,-} composed of the four nucleotides plus the gap character "-". The probabilities are approximated by the observed frequencies (e.g. $p_{A}^{i}$ is the frequency of the character A in column *i *divided by the number of sequences in the alignment). The normalized Shannon entropy of an alignment A is then defined as the sum of the Shannon entropies of the individual columns divided by the length of the alignment denoted by *L*:

(21) $$H=-\frac{1}{L}\sum_{i}^{L}\sum_{j\in \Sigma }p_{j}^{i}\log_{2}p_{j}^{i}$$

**Figure 1:**
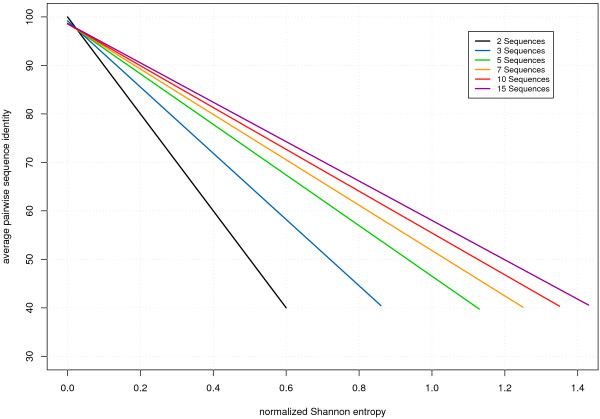
**Relation between the average pairwise sequence identity and the normalized Shannon entropy.** The Shannon entropy is used as measure for information content contained in an alignment throughout this paper. It depends on the average pairwise identity and the number of sequences in the alignment. The lines shown are regression lines for the nearly exact linear relationship (*R*^2 ^> 0.99) between Shannon Entropy and the mean pairwise identity.

Although it is convenient to use this measure, most people are more familiar with the API. Fig. 1 shows the relation of the API and the Shannon entropy for alignments with different number of sequences.

In order to assess and compare the performance of the various strategies, we perform receiver operating characteristic (ROC) curve analysis. A ROC curve [60] is a plot of the true positive rate (sensitivity) versus the false positive rate (1-specificity), while varying the discrimination threshold of a scoring classifier. The more a ROC curve is shifted to the upper left corner of the plot, the better the discrimination is. The area under the ROC curve (AUC) is a single scalar value ranging from 0 to 1 representing the overall discrimination capability of a method. A random classifier has an AUC value around 0.5, while perfect classification is indicated by an AUC value of 1.

## Results and Discussion

### Overview

**Figure 2:**
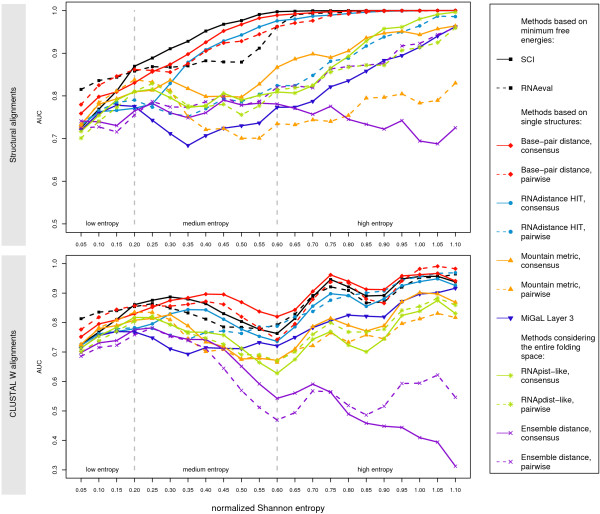
Results of the benchmark. AUC values (area under the ROC curve) are shown as general performance measure for different methods, different alignment sets and different regions of information content. Also refer to Tab. 2.

The results of the benchmark are summarized in Tab. 2 and Fig. 2. The test set was binned by entropy and for each bin we calculated the average AUC as overall performance measure for each method. In Table 2, we additionally give the sensitivity of each method for a given specificity of 95%. In other words, this number is the percentage of correctly identified conserved structures at a false positive rate of 5%, a somewhat more practical measure than the AUC. Almost all methods can be applied in a pairwise comparison manner and as a comparison of single structures to a consensus structure/energy. We will simply refer to these cases as 'pairwise' and 'consensus'.

**Table 2:** Comparison of different strategies

		Structural						CLUSTAL W					
			
Method	Variant	Low		Medium		High		Low		Medium		High	
Energy based	SCI	0.79	0.32	**0.95**	**0.70**	**1.00**	**1.00**	0.79	0.31	0.80	**0.42**	0.90	0.72
	RNAeval	0.82	**0.43**	0.86	0.45	**1.00**	0.99	0.82	0.42	0.76	0.32	0.90	0.68
Base-pair distance	consensus	0.80	0.28	0.93	0.56	**1.00**	0.99	0.79	0.27	**0.85**	0.40	**0.92**	**0.79**
	pairwise	0.83	0.28	0.90	0.54	0.99	0.98	**0.83**	0.27	0.81	0.40	0.90	0.78
Mountain metric	consensus	0.78	0.34	0.82	0.38	0.92	0.63	0.78	0.34	0.73	0.29	0.80	0.41
	pairwise	0.79	0.29	0.75	0.33	0.76	0.34	0.79	0.29	0.73	0.30	0.75	0.34
Tree editing	consensus, full	0.77	0.32	0.88	0.44	0.99	0.95	0.77	0.32	0.77	0.31	0.86	0.60
	consensus, HIT	0.76	0.30	0.89	0.46	0.99	0.97	0.76	0.28	0.78	0.33	0.87	0.60
	consensus, coarse grained	0.71	0.22	0.81	0.34	0.95	0.73	0.72	0.21	0.74	0.26	0.83	0.45
	consensus, w. coarse grained	0.74	0.26	0.84	0.36	0.98	0.88	0.74	0.25	0.73	0.28	0.82	0.46
	pairwise, full	0.78	0.31	0.77	0.36	0.88	0.63	0.78	0.31	0.75	0.34	0.87	0.56
	pairwise, HIT	0.77	0.27	0.77	0.36	0.90	0.66	0.76	0.26	0.76	0.34	0.89	0.63
	pairwise, coarse grained	0.72	0.16	0.68	0.23	0.74	0.24	0.72	0.16	0.68	0.22	0.78	0.30
	pairwise, w. coarse grained	0.76	0.23	0.71	0.28	0.81	0.41	0.75	0.15	0.71	0.23	0.82	0.35
	pairwise, MiGaL-Layer 0	0.62	0.07	0.61	0.07	0.67	0.06	0.62	0.07	0.60	0.06	0.66	0.04
	pairwise, MiGaL-Layer 1	0.74	0.27	0.68	0.24	0.77	0.33	0.74	0.27	0.68	0.24	0.76	0.33
	pairwise, MiGaL-Layer 2	0.74	0.23	0.70	0.29	0.82	0.42	0.73	0.22	0.69	0.27	0.78	0.37
	pairwise, MiGaL-Layer 3	0.76	0.27	0.71	0.30	0.84	0.49	0.75	0.26	0.71	0.29	0.82	0.47
Ensemble distance	consensus	0.64	0.32	0.61	0.15	0.72	0.25	0.63	0.31	0.60	0.14	0.70	0.24
	pairwise	0.65	0.42	0.61	0.15	0.72	0.26	0.65	0.32	0.61	0.30	0.72	0.31
Mountain metric using base-pair probabilities	consensus	0.48	0.17	0.58	0.27	0.65	0.40	0.50	0.18	0.56	0.24	0.61	0.28
	pairwise	0.78	0.32	0.75	0.34	0.76	0.31	0.79	0.32	0.72	0.30	0.74	0.31
RNApdist-like	consensus	0.76	0.28	0.79	0.37	0.89	0.44	0.76	0.27	0.73	0.30	0.74	0.25
	pairwise	0.75	0.25	0.78	0.36	0.86	0.45	0.75	0.24	0.73	0.28	0.78	0.30

Low, Medium and High refer to the same information content categories as shown in Fig. 2. Each column consists of two numbers: Left: average AUC, Right: sensitivity at 5% false positive rate. Bold numbers indicate the most accurate method in each category.

As a main result one can note that over all entropy ranges and for both the structural and sequence based alignments, either an energy based method (SCI/RNAeval) or the base-pair distance performs best. These methods are followed by the tree editing methods based on RNAdistance. MiGaL based tree editing, mountain metric and ensemble methods perform significantly worse.

### SCI/RNAeval and base-pair distance

In general, the SCI shows the best overall discrimination power on the structural alignments. On the medium and high entropy sets it apparently makes use of the large number of consistent/compensatory mutations that are explicitly considered in the SCI through the RNAalifold consensus energy that contains a covariation score. The use of the covariation scoring model in RNAalifold does improve the discrimination capability of the SCI significantly compared to a version where the covariation score was turned off (data not shown).

Only on the low entropy set that contains highly conserved alignments with little evolutionary information the SCI is outperformed by the RNAeval and base-pair distance measures. In cases with only a few structural changes, the base-pair distance, which considers the exact position of pairs, seems to be more sensitive than the SCI that uses the folding energy as abstraction of the structure.

Interestingly, the clear winner in the low entropy set is the RNAeval method that, similar to the SCI, also uses the folding energy instead of the structure itself. Still, it performs significantly better (*p*-values < 0.001) than the SCI. The SCI and the RNAeval approach operate on two different scales. While the SCI is bounded below by 0, the RNAeval approach is bounded above by 1, which causes favoring of two extreme cases. In the case of the SCI an alignment with loads of compensatory and consistent mutations will yield a SCI above 1 due to the covariance score. The RNAeval approach will give at most 1 as compensatory and consistent mutations are not specially rewarded. In the case of an alignment of sequences that do not share a common fold the SCI will be 0, while the RNAeval approach will yield a value below 0 as the evaluation of a sequence forced to fold into a structure that is not likely to be adopted by that sequence will give positive energy values. Hence, in the case of the SCI we are dealing with a better dispersion of positive examples, and vice versa in the RNAeval approach with a better dispersion of negative examples.

The overall trend looks slightly different on the CLUSTAL W generated alignments. The SCI loses discrimination power and the base-pair distance performs equally well or, in most cases, even better. So it seems that the base-pair distance is more robust against alignment errors than the SCI.

Another difference between the results for the structural and CLUSTAL W sets is the overall shape of the curves in Fig. 2. For the structural alignments, the classification power increases with increasing information content. This trend is of course entirely expected, and it is also visible for the CLUSTAL W alignments. However, there are two marked valleys at about 0.6 and 0.9 Shannon entropy. The first one is caused by a prevalence of pairwise alignments with low sequence identity. An average pairwise identity of 60% to 65% or below is considered as critical with regard to secondary structures for alignments generated solely on sequence information [26]. This results in a relatively low discrimination capability in this region. As soon as low identity pairwise alignments do not constitute the majority of instances in a bin, the predictive power rises again. The second performance drop is again caused by prevalence of alignments with low sequence identity, in this case alignments with three sequences.

### Tree editing

**Figure 3:**
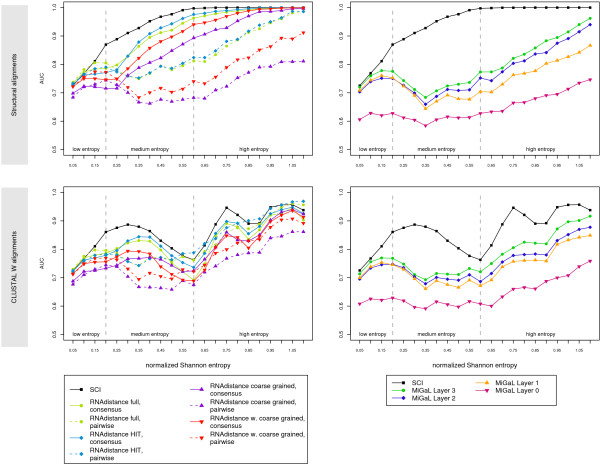
**Detailed benchmark results for the tree editing methods.** AUC values are shown for all variants of the tree editing methods, including different algorithms and abstraction levels. Also refer to Tab. 2.

The best tree editing approach (the consensus approach using the HIT representation), in general shows weaker performance than the SCI on both the structural and the CLUSTAL W generated data sets. Detailed results for all tree editing methods are shown in Fig. 3. There is a clear hierarchy among tree editing approaches. An abstraction of structural details in the representation is accompanied with a loss in discrimination power, which is especially well pronounced on the structural data set. Tree editing using the full and HIT representations, which encode a RNA secondary structure without any loss of information, give best results, while the coarse grained approach which is abstracting at most shows the weakest performance.

The weighted coarse-grained approach maintains a higher level of structural information than the coarse-grained representation and therefore generally performs better. The use of different costs for the tree editing operations has significant influences on the discrimination power of the methods. Tree editing distances of the coarse-grained and weighted coarse-grained representations were calculated using the cost matrix of the *Vienna RNA *package and the costs initially proposed by Shapiro [42]. Although the editing costs are in both cases chosen more ore less arbitrarily, the weighted coarse-grained approach using the *Vienna RNA *package costs performs significantly better or at least equally well on both structural and CLUSTAL W generated alignments than the weighted coarse-grained approach using Shapiro's costs (data not shown).

As MiGaL makes use also of the nucleotide sequence and not secondary structures alone, we evaluated MiGaL only in pairwise comparisons. Also for the MiGaL methods, we observe the trend that the more information is encoded in a representation or layer, respectively, the better the discrimination capability. However, despite its more sophisticated algorithm, MiGaL performs worse than the simpler tree editing algorithms of the *Vienna RNA *package.

Tree editing is the only method that can be applied *per se *to sequences of unequal length, and is hence not subjected to the alignment quality. This seems to be an advantage of this method. However, this only holds for pairwise comparisons as the calculation of a consensus structure is dependent on a given alignment. Since the consensus approaches show much better performance than their pairwise counterparts on structural alignments, and at least comparable results on CLUSTAL W generated alignments, the advantage of alignment independent pairwise comparisons is questionable.

### Mountain metric

The mountain metric shows the weakest performance of all methods that are based on single structures. This trend becomes even worse when using base-pairing probabilities. Although the mountain representation allows easy comparison of RNA structures by visual examination, when put to formalism by the mountain metric this approach fails. The weak performance indicates that the difference in the mountain functions of closely related RNA molecules is in many cases in the range of differences one obtains by comparing non-related structures.

### Ensemble methods

In principle, secondary structure predictions that take into account the whole thermodynamical ensemble of the folded RNA hold more information than the mere MFE structure. However, we observe that this does not translate into improved detection performance of conserved RNA structures (Fig. 2, Table 2). The ensemble distance shows only moderate performance on structural alignments, and fails completely on CLUSTAL W generated alignments. It seems that taking into account sub-optimal base-pairs only adds noise to the comparison and blurs the signal instead of improving it.

The extreme sensitivity to alignment errors can be explained by the fact that each probability of each possible base-pair of one sequence has to be compared to the corresponding probability of the other sequences or the consensus, respectively. A base-pair present in one ensemble that does not have a counterpart in the other ensembles adds its full squared probability to the distance.

The RNApdist-like methods show best overall performance of the ensemble based methods. This is consistent with the observations above, since the RNApdist method only considers a condensed and thus lessnoisy version of the full pair-probability matrix.

### Consensus versus pairwise comparison

In general, one can observe that methods based on the comparison to a consensus structure perform better than methods based on pairwise comparisons only. The consensus structure predicted by RNAalifold which is usually more accurate than single structures prediction, improves the discrimination power significantly. There are two exceptions: In the case of the ensemble methods and in the low entropy test-set, the trend is reversed with pairwise methods performing better than their consensus variant.

In the case of the ensemble methods, this is apparently due to the way base-pairing probabilities are calculated by RNAfold and RNAalifold. For single sequence there are no special rules for two bases to form a base-pair, they just have to belong to the set of valid base-pairs. RNAfold can therefore assign a base-pair probability to each valid base-pair. On the alignment level this is more complicated as we are dealing with columns of nucleotides rather than with single nucleotides. In the RNAalifold algorithm, only those column pairs in which at least 50% of the sequences can form a base-pair are used in the computation. In the case of the consensus comparison approach there may be many base-pairing probabilities in the single sequences that do not have a consensus counterpart.

Also in the low entropy range, which is dominated by alignments with little sequence variation, pairwise comparison approaches show better discrimination capability than their consensus counterparts. Here, there is almost no additional mutational information that could give RNAalifold an advantage over RNAfold on single sequences.

### Other methods

As both ddbRNA and MSARi show limitations to the data sets that can be applied, we evaluated both methods only on appropriate subsets of our test set. In case of ddbRNA these are pairwise and three-way alignments, and in case of MSARi 10-way and 15-way alignments.

**Figure 4:**
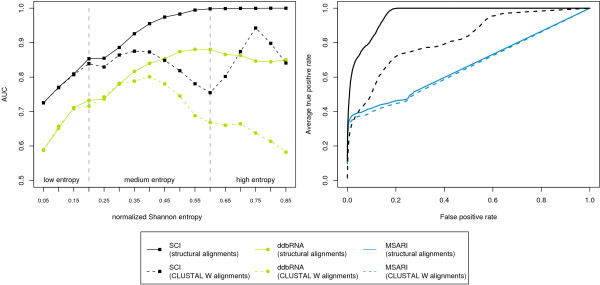
**Performance of the MSARi and ddbRNA algorithms.Left: **AUC values for ddbRNAin comparison to the SCI. Only pairwise and three-way alignments were considered. **Right**: ROC curves of 10- and 15-way alignments for MSARi in comparison to the SCI.

In this study we use ddbRNA to evaluate the number of compensatory mutations per length as a measure of evolutionary conservation of structure. The ddbRNA approach shows only moderate discrimination capability and performs significantly worse than the SCI on both structural and CLUSTAL W generated alignments (Fig. 4). ddbRNA is extremely sensitive to the alignment quality as the detected stems must be present in all sequences of an alignment.

As MSARi implements a strategy that compensates slight mis-alignments, the results are almost identical for structural and CLUSTAL W generated alignments, but it shows significant lower discrimination capability than most other methods tested in this paper, e.g. the SCI as shown in Fig. 4. The shape of the ROC curves for MSARi indicates that only a few conserved instances are detected as truly conserved. They are assigned very low *p*-values and it is not likely to find false positive examples at this low level. However, a large fraction of conserved instances is not considered to be conserved and is assigned a *p*-value of 1.

**Figure 5:**
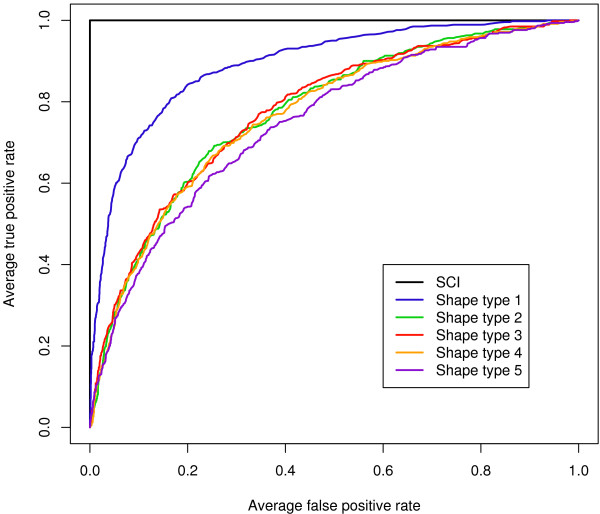
**Performance of the RNAshapes based method.** ROC curves are shown for different abstraction levels on a test set of 461 five-way alignments of tRNAs from the structural data set.

Due to the exponential growth of the shape space with the length of the sequence and the resulting computational costs, we evaluated the RNAshapes approach as a proof of concept only on a small set of tRNAs. Although this method shows clear discrimination capability, it is far below the performance of the SCI which is able to perfectly separate this specific tRNA test set (Fig. 5). The observation that the shape type 1 (lowest level of abstraction) performs significantly better than the shape type 5 (highest level of abstraction) is consistent with the observations that increasing abstraction of detailed structural information is related to a loss in discrimination power.

### Correlation of methods

**Figure 6:**
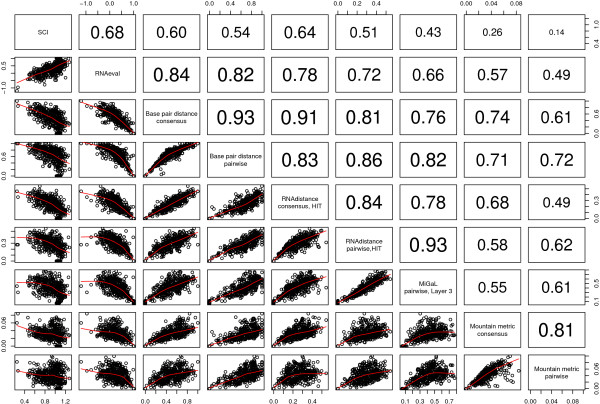
**Correlation of selected methods.** Lower triangular matrix scatter plots of the different scores with local regression indicted by red lines. Upper triangular matrix displays the corresponding Pearson correlation coefficients. Data points are shown for structural alignments in an entropy range from 0.4 to 0.6 and a GC content limited to an interval of 0.48 to 0.52.

We have tested a variety of different methods in order to measure the same property, namely structural conservation. A question that is still open is whether all these methods essentially detect the same features or focus on different aspects of the conserved structures. To get some clues on this question, we investigated the correlation between selected measures (Fig. 6). All methods correlate statistically significantly (*p *< 0.001) with each other on the tested subset. The degree of correlation varies, however. Not surprisingly, among the highest correlations (correlation coefficient 0.93) are the two tree editing methods using the HIT representation and MiGaL Layer 3, as they act both on trees of full structural detail. The base-pair distance is also highly correlated with the tree editing methods. The SCI shows the highest correlation to RNAeval (0.68), which again does not come unexpected, as both measures are based on folding energies. However, the relatively high degree of correlation between SCI/RNAeval and the other methods is remarkable. RNAeval, for example, has the same degree of correlation to the pairwise base-pair distance (0.82) as the pairwise base-pair distance to the pairwise RNAdistance measure. This shows that also SCI/RNAeval, methods that actually do not regard the structure, effectively measure it. This seems noteworthy, as the name "Structure conservation index" has been criticized in the past of being misleading because the SCI does not measure structural conservation explicitly.

### Dependence on base composition

All scores used in this study are normalized with respect to sequence length and the number of sequences in the alignment. In principle, all our methods should also be independent of the base composition. The energy based methods SCI and RNAeval compare folding energies in a way that the absolute value of the free energy (which is clearly dependent on the GC content) is also normalized. All other methods, except tree editing using MiGaL with Layer 3, do not even explicitly consider the sequence but act on the predicted structure only. Although all methods should be normalized for base composition by construction, we still investigated how they are affected by the GC content.

**Figure 7:**
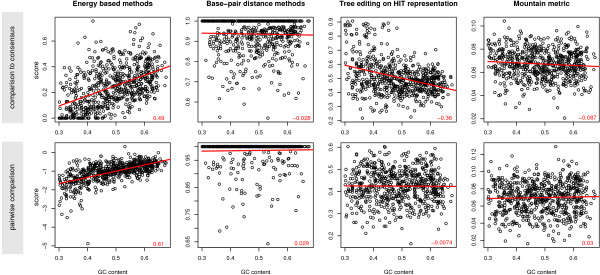
**Dependency on nucleotide composition of selected methods.** The scores of a subset of randomized pairwise alignments of tRNAs in an entropy range from 0.4 to 0.6 are plotted against the average GC content of the sequences in the alignment. Correlation coefficients are indicated in red at the bottom of each plot.

The somewhat surprising results are shown in Fig. 7. While pairwise tree editing, base-pair distance and mountain metric approaches do not show any significant correlation to the GC content, energy based methods and tree editing using a consensus structure derived by RNAalifold show high correlation. The consensus base-pair distance method shows little correlation, but correlation increases slightly when moving to higher entropy ranges (data not shown).

These results suggest that the observed GC-dependence is mainly a consequence of using a RNAalifold consensus structure. In the case of the SCI, this is easiest to understand. The SCI is the ratio of the consensus energy and the mean of the single sequence energies. Both components are functions of the base composition, with higher GC content resulting in lower free energies. Although consensus predictions use the same energy model as single sequence predictions, the additional constraints imposed by folding several sequences together result in a slightly different GC dependence. Similar effects seem to be responsible for the GC dependence of the RNAeval measure and the consensus based tree editing measures.

For the purpose of this study, the GC dependence does not directly affect the results due to the design of our benchmark. The positive and negative test set contains sequences with the same base composition. However, for practical reasons when considering these measures in RNA gene finding algorithms this effect is of relevance. The GC dependence of the SCI seems to be the main reason why the RNAz program shows a small bias towards GC rich regions [61].

### Statistical significance of the scores

In this study we compared the different methods on the basis of their ability to discriminate between alignments containing true conserved structures and random controls. While this approach gives us information on the performance of the methods relative to each other, none of the scores used in this study (except the MSARi *p*-value) is normalized for sequence diversity. Alignments with 100% sequence identity get, by definition, the highest score of perfect structure conservation. For the purpose of detecting evolutionary conserved structures this is of little help. Ideally, one would like to answer the question of whether there is an unusually conserved structure in an alignment despite the given sequence diversity.

This problem can be addressed in different ways. The optimal solution is to devise a direct statistical model as in the case of MSARi. However, this seems only feasible if one considers a simplified score like the base-pair derived score in MSARi. It seems impossible to analytically derive the background distribution of a more complex score like the SCI, since it depends on complex folding algorithms that cannot be modeled directly.

As an alternative, machine learning algorithms can be used. In the case of RNAz, the dependence of the SCI on the number of sequences and the average pairwise identity is trained on a large test set of known ncRNAs and random alignments.

Yet another possibility is to derive the background distribution empirically for each alignment under test. This approach is used by AlifoldZ, which calculates a *z*-score by comparing the score of the original alignment to the score distribution of randomized alignments.

This last method is computationally demanding, but has the advantage that it can be applied to any score without modification.

### Availability

We have set up a web-server that calculates relevant scores used in this study for a given alignment and assesses the statistical significance by calculating a *z*-score and an empirical *p*-value. The web server can be accessed under .

## Conclusion

The aim of this work was to find the most effective ways to detect evolutionarily conserved RNA structures in sequence alignments. A few methods and algorithms have been proposed previously. Here, we devised a series of novel measures and evaluated their performance systematically on a large test set of known conserved RNA structures.

As the most accurate measures we could identify the folding energy based "structure conservation index" and a measure based on the base-pair-distance structure metric. Interestingly, these two are among the simplest methods tested and generally outperform all of the more sophisticated methods. Only the methods based on tree editing distances could compete to some degree with the SCI/base-pair distance. Here we can note that more complex tree representations show better performance than simplified "coarse grained" abstractions. However, more sophisticated algorithms like MiGaL do not give better results than the basic algorithms as implemented in the *Vienna RNA *package. All other methods show only very poor performance and do not appear to be a reasonable choice in any "real-life" application. Among these methods we have to list the mountain metric, all methods based on structure ensembles and also the ddbRNA and MSARi algorithm.

As a general trend we could observe that the measures relying on a consensus structure prediction by the RNAalifold algorithm have clear advantage over methods that only use single sequence structure predictions.

All these results are fairly consistent over all tested alignments with one notable exception. For highly conserved sequences the RNAeval approach based on pairwise folding energy comparisons shows the highest accuracy and all other measures, including the SCI, perform significantly worse.

Taken together we can conclude that the simple methods based on either folding energies or base-pair distance are the methods of choice. Although the SCI was the only method that was tested when RNAz was first published, our results clearly show that this was a reasonable choice. An interesting new aspect is the GC dependence of the SCI that we observed here. This makes it necessary to consider base composition when evaluating the statistical significance of the SCI, for example by including the GC content as an additional classifier in the RNAz machine learning algorithm. This can be expected to increase the specificity of the program.

Another result which has practical implications is the fact that the SCI performs poorly on highly conserved sequences. The RNAeval method turned out to be significantly better and might help to improve ncRNA gene prediction under these particularly difficult conditions.

The ever-growing pace of current genome sequencing projects confronts current RNA gene finders with new problems. Having sequences of dozens or even hundreds of species, the paradigm of detecting conserved structures will change. Only a few extraordinarily conserved RNAs like tRNAs or rRNAs will show a signal of structure conservation across the whole phylogeny. The next generation of RNA gene finders will have to deal with the problem of finding lineage specific and evolving structures. The strategies presented here can be the basis of algorithms that find sub-groups of related structures or detect outliers of mis-aligned sequences. We plan to enhance our programs RNAz and AlifoldZ with such capabilities. The results obtained here guide such efforts as they clearly show which measures are worth considering and which should be avoided.

## Methods

All results presented in this paper are based on the *BRAliBase 2.1 *data set [27]. It consists of 18,990 structural alignments of 36 RNA families. Alignments are divided into subsets of alignments with 2, 3, 5, 7, 10, and 15 sequences (see additional File 1). For each alignment in *BRAliBase 2.1 *a corresponding sequence based alignment using CLUSTAL W, version 1.83, with standard settings was generated. Negative controls (i.e. alignments without naturally evolved secondary structure) were generated by shuffling using shuffle-aln.pl [5] with option "--conservative2". This shuffling procedure maintains the gap pattern and only columns with the same degree of conservation are shuffled. This results in randomized alignments of the same length, the same number of sequences, the same nucleotide composition, the same overall conservation, the same local conservation and the same gap pattern. For each alignment in the original *BRAliBase 2.1 *and CLUSTAL W data set, respectively, five randomized alignments were generated for subsequent ROC analysis.

Alignments in both data sets were split according to their normalized Shannon entropy (equation 21) in sub sets with a bin size of 0.05. For determination of a minimal sample size, we followed the strategy proposed by Hanley & McNeil [60]. A minimal sample size of 200 positive and 200 negative instances seems to yield reasonable results (i.e. low standard error). The relative gain in a lower standard error is small when moving to a higher sample size. To statistically assess the significance of the difference of two AUC values we then used the non-parametric method by DeLong [62]. Calculation of AUC values was done using the R statistical package, version 2.5.1, and the ROCR package [63].

As many methods can only be applied to structures of equal length, RNA sequences without gap characters were folded using RNAfold. The alignment of the sequences was then used to reintroduce gaps into structures (denoted simply as .) or to adjust the position of base-pairs when using base-pairing probabilities.

### Programs and options used

The following program versions and options were used from the *Vienna RNA *package, version 1.6.5: RNAfold for calculations of MFE structures and base-pair probabilities of single sequences with options -p -d2. RNAalifold for calculation of consensus structures and consensus base-pair probabilities with options -p -d2. RNAeval for energy evaluations of a sequence in a given secondary structure. RNAdistance for calculation of base-pair distances with option -DP and tree editing distances with options -Dfhwc and additional option -S when calculations are done using Shapiro's cost matrix.

Other programs not part of the *Vienna RNA *package: RNAshapes version 2.1.1 with options -p-t [1|2|3|4|5]. migal version 2 with options -M --memory 1000. ddbRNA with standard options. MSARi with standard options.

## Authors' contributions

All authors contributed to the design of the study and the interpretation of the results. ARG carried out the analysis. ARG and SW wrote the manuscript. All authors read and approved the final manuscript.

## Supplementary Material

Additional file 1 Overview of the BRAliBase 2.1 dataset. Overview of the *BRAliBase 2.1 *data set. The number of the alignments in the different entropy bins are shown. The red line indicates the minimal threshold of positive instances we used to obtain reasonable significance levels in the ROC analysis. Bins below this threshold were not considered. Click here for file

## References

[B1] Bompfünewerer A, Flamm C, Fried C, Fritzsch G, Hofacker I, Lehmann J, Missal K, Mosig A, Müller B, Prohaska S, Stadler B, Stadler P, Tanzer A, Washietl S, Witwer C (2005). Evolutionary patterns of non-coding RNAs. Theor Biosci.

[B2] Mignone F, Gissi C, Liuni S, Pesole G (2002). Untranslated regions of mRNAs. Genome Biol.

[B3] Rivas E, Eddy SR (2001). Noncoding RNA gene detection using comparative sequence analysis. BMC Bioinformatics.

[B4] Pedersen JS, Bejerano G, Siepel A, Rosenbloom K, Lindblad-Toh K, Lander ES, Kent J, Miller W, Haussler D (2006). Identification and classification of conserved RNA secondary structures in the human genome. PLoS Comput Biol.

[B5] Washietl S, Hofacker IL (2004). Consensus folding of aligned sequences as a new measure for the detection of functional RNAs by comparative genomics. J Mol Biol.

[B6] Hofacker IL, Fekete M, Stadler PF (2002). Secondary structure prediction for aligned RNA sequences. J Mol Biol.

[B7] Washietl S, Hofacker IL, Stadler PF (2005). Fast and reliable prediction of noncoding RNAs. Proc Natl Acad Sci USA.

[B8] Coventry A, Kleitman DJ, Berger B (2004). MSARi: multiple sequence alignments for statistical detection of RNA secondary structure. Proc Natl Acad Sci USA.

[B9] di Bernardo D, Down T, Hubbard T (2003). ddbRNA: detection of conserved secondary structures in multiple alignments. Bioinformatics.

[B10] Backofen R, Bernhart SH, Flamm C, Fried C, Fritzsch G, Hackermuller J, Hertel J, Hofacker IL, Missal K, Mosig A, Prohaska SJ, Rose D, Stadler PF, Tanzer A, Washietl S, Will S (2007). RNAs everywhere: genome-wide annotation of structured RNAs. J Exp Zoolog B Mol Dev Evol.

[B11] Mourier T, Carret C, Kyes K, Christodoulou Z, Gardner P, Jeffares DC, Pinches R, B B, Berriman M, Griffiths-Jones S, Ivens A, Newbold C, Pain A (2008). Genome wide discovery and verification of novel structured RNAs in Plasmodium falciparum. Genome Research.

[B12] Stark A, Lin MF, Kheradpour P, Pedersen JS, Parts L, Carlson JW, Crosby MA, Rasmussen MD, Roy S, Deoras AN, Ruby JG, Brennecke J, Curators HF, Project BD, Hodges E, Hinrichs AS, Caspi A, Paten B, Park SW, Han MV, Maeder ML, Polansky BJ, Robson BE, Aerts S, van Helden J, Hassan B, Gilbert DG, Eastman DA, Rice M, Weir M, Hahn MW, Park Y, Dewey CN, Pachter L, Kent WJ, Haussler D, Lai EC, Bartel DP, Hannon GJ, Kaufman TC, Eisen MB, Clark AG, Smith D, Celniker SE, Gelbart WM, Kellis M, Crosby MA, Matthews BB, Schroeder AJ, Sian Gramates L, St Pierre SE, Roark M, Wiley KL, Kulathinal RJ, Zhang P, Myrick KV, Antone JV, Gelbart WM, Carlson JW, Yu C, Park S, Wan KH, Celniker SE (2007). Discovery of functional elements in 12 Drosophila genomes using evolutionary signatures. Nature.

[B13] Rose D, Hackermueller J, Washietl S, Reiche K, Hertel J, Findeiss S, Stadler PF, Prohaska SJ (2007). Computational RNomics of Drosophilids. BMC Genomics.

[B14] Steigele S, Huber W, Stocsits C, Stadler PF, Nieselt K (2007). Comparative analysis of structured RNAs in S. cerevisiae indicates a multitude of different functions. BMC Biol.

[B15] Washietl S, Hofacker IL, Lukasser M, Hüttenhofer A, Stadler PF (2005). Mapping of conserved RNA secondary structures predicts thousands of functional noncoding RNAs in the human genome. Nat Biotechnol.

[B16] Missal K, Zhu X, Rose D, Deng W, Skogerbo G, Chen R, Stadler PF (2006). Prediction of structured non-coding RNAs in the genomes of the nematodes Caenorhabditis elegans and Caenorhabditis briggsae. J Exp Zoolog B Mol Dev Evol.

[B17] Missal K, Rose D, Stadler PF (2005). Non-coding RNAs in Ciona intestinalis. Bioinformatics.

[B18] Uzilov AV, Keegan JM, Mathews DH (2006). Detection of non-coding RNAs on the basis of predicted secondary structure formation free energy change. BMC Bioinformatics.

[B19] Torarinsson E, Sawera M, Havgaard JH, Fredholm M, Gorodkin J (2006). Thousands of corresponding human and mouse genomic regions unalignable in primary sequence contain common RNA structure. Genome Res.

[B20] Weinberg Z, Barrick JE, Yao Z, Roth A, Kim JN, Gore J, Wang JX, Lee ER, Block KF, 'Sudarsan N, Neph S, Tompa M, Ruzzo WL, Breaker RR (2007). Identification of 22 candidate structured RNAs in bacteria using the CMfinder comparative genomics pipeline. Nucleic Acids Res.

[B21] Yao Z, Barrick J, Weinberg Z, Neph S, Breaker R, Tompa M, Ruzzo WL (2007). A Computational Pipeline for High-Throughput Discovery of cis-Regulatory Noncoding RNA in Prokaryotes. PLoS Comput Biol.

[B22] Miller W, Rosenbloom K, Hardison RC, Hou M, Taylor J, Raney B, Burhans R, King DC, Baertsch R, Blankenberg D, Kosakovsky Pond SL, Nekrutenko A, Giardine B, Harris RS, Tyekucheva S, Diekhans M, Pringle TH, Murphy WJ, Lesk A, Weinstock GM, Lindblad-Toh K, Gibbs RA, Lander ES, Siepel A, Haussler D, Kent WJ (2007). 28-Way vertebrate alignment and conservation track in the UCSC Genome Browser. Genome Res.

[B23] Babak T, Blencowe BJ, Hughes TR (2007). Considerations in the identification of functional RNA structural elements in genomic alignments. BMC Bioinformatics.

[B24] Will S, Reiche K, Hofacker IL, Stadler PF, Backofen R (2007). Inferring noncoding RNA families and classes by means of genome-scale structure-based clustering. PLoS Comput Biol.

[B25] Freyhult EK, Bollback JP, Gardner PP (2007). Exploring genomic dark matter: a critical assessment of the performance of homology search methods on noncoding RNA. Genome Res.

[B26] Gardner PP, Wilm A, Washietl S (2005). A benchmark of multiple sequence alignment programs upon structural RNAs. Nucleic Acids Res.

[B27] Wilm A, Mainz I, Steger G (2006). An enhanced RNA alignment benchmark for sequence alignment programs. Algorithms Mol Biol.

[B28] Andersen ES, Lind-Thomsen A, Knudsen B, Kristensen SE, Havgaard JH, Torarinsson E, Larsen N, Zwieb C, Sestoft P, Kjems J, Gorodkin J (2007). Semiautomated improvement of RNA alignments. RNA.

[B29] Collins LJ, Moulton V, Penny D (2000). Use of RNA secondary structure for studying the evolution of RNase P and RNase MRP. J Mol Evol.

[B30] Caetano-Anolles G (2002). Evolved RNA secondary structure and the rooting of the universal tree of life. J Mol Evol.

[B31] Holmes I (2004). A probabilistic model for the evolution of RNA structure. BMC Bioinformatics.

[B32] Zuker M, Stiegler P (1981). Optimal computer folding of large RNA sequences using thermodynamics and auxiliary information. Nucleic Acids Res.

[B33] Hofacker IL, Fontana W, Stadler PF, Bonhoeffer LS, Tacker M, Schuster P (1994). Fast folding and comparison of RNA secondary structures. Monatsh Chem.

[B34] Mathews DH, Turner DH (2006). Prediction of RNA secondary structure by free energy minimization. Curr Opin Struct Biol.

[B35] Flamm C, Fontana W, Hofacker IL, Schuster P (2000). RNA folding at elementary step resolution. RNA.

[B36] Dowell RD, Eddy SR (2004). Evaluation of several lightweight stochastic context-free grammars for RNA secondary structure prediction. BMC Bioinformatics.

[B37] Knudsen B, Hein J (2003). Pfold: RNA secondary structure prediction using stochastic context-free grammars. Nucleic Acids Res.

[B38] Do CB, Woods DA, Batzoglou S (2006). CONTRAfold: RNA secondary structure prediction without physics-based models. Bioinformatics.

[B39] Hogeweg P, Hesper B (1984). Energy directed folding of RNA sequences. Nucleic Acids Res.

[B40] Moulton V, Zuker M, Steel M, Pointon R, Penny D (2000). Metrics on RNA secondary structures. J Comput Biol.

[B41] Shapiro BA (1988). An algorithm for comparing multiple RNA secondary structures. Comput Appl Biosci.

[B42] Shapiro BA, Zhang KZ (1990). Comparing multiple RNA secondary structures using tree comparisons. Comput Appl Biosci.

[B43] Fontana W, Konings DA, Stadler PF, Schuster P (1993). Statistics of RNA secondary structures. Biopolymers.

[B44] Allali J, Sagot MF (2005). A new distance for high level RNA secondary structure comparison. IEEE/ACM Transactions on Computational Biology and Bioinformatics.

[B45] Allali J, Sagot MF (2005). A multiple graph layers model with application to RNA secondary structures comparison. String Processing and Information Retrieval.

[B46] McCaskill JS (1990). The equilibrium partition function and base pair binding probabilities for RNA secondary structure. Biopolymers.

[B47] Huynen MA, Perelson A, Vieira WA, Stadler PF (1996). Base pairing probabilities in a complete HIV-1 RNA. J Comput Biol.

[B48] Bonhoeffer S, McCaskill JS, Stadler PF, Schuster P (1993). RNA multi-structure landscapes. A study based on temperature dependent partition functions. Eur Biophys J.

[B49] Giegerich R, Voss B, Rehmsmeier M (2004). Abstract shapes of RNA. Nucleic Acids Res.

[B50] Voss B, Giegerich R, Rehmsmeier M (2006). Complete probabilistic analysis of RNA shapes. BMC Biol.

[B51] Steffen P, Voss B, Rehmsmeier M, Reeder J, Giegerich R (2006). RNAshapes: an integrated RNA analysis package based on abstract shapes. Bioinformatics.

[B52] Sankoff D (1985). Simultaneous Solution of the RNA Folding, Alignment and Protosequence Problems. SIAM Journal on Applied Mathematics.

[B53] Klein RJ, Eddy SR (2003). RSEARCH: finding homologs of single structured RNA sequences. BMC Bioinformatics.

[B54] Wolf M, Achtziger M, Schultz J, Dandekar T, Müller T (2005). Homology modeling revealed more than 20,000 rRNA internal transcribed spacer 2 (ITS2) secondary structures. RNA.

[B55] Jiang T, Lin G, Ma B, Zhang K (2002). A General Edit Distance between RNA Structures. J Comp Biol.

[B56] Hochsmann M, Toller T, Giegerich R, Kurtz S (2003). Local Similarity in RNA Secondary Structures. csb.

[B57] Liu N, Wang T (2006). A method for rapid similarity analysis of RNA secondary structures. BMC Bioinformatics.

[B58] Thompson JD, Higgins DG, Gibson TJ (1994). CLUSTAL W: improving the sensitivity of progressive multiple sequence alignment through sequence weighting, position-specific gap penalties and weight matrix choice. Nucleic Acids Res.

[B59] Yao Z, Weinberg Z, Ruzzo WL (2006). CMfinder-a covariance model based RNA motif finding algorithm. Bioinformatics.

[B60] Hanley JA, McNeil BJ (1982). The meaning and use of the area under a receiver operating characteristic (ROC) curve. Radiology.

[B61] Washietl S, Pedersen JS, Korbel JO, Stocsits C, Gruber AR, Hackermüler J, Hertel J, Lindemeyer M, Reiche K, Tanzer A, Ucla C, Wyss C, Antonarakis SE, Denoeud F, Lagarde J, Drenkow J, Kapranov P, Gingeras TR, Guigó R, Snyder M, Gerstein MB, Reymond A, Hofacker IL, Stadler PF (2007). Structured RNAs in the ENCODE selected regions of the human genome. Genome Res.

[B62] DeLong ER, DeLong DM, Clarke-Pearson DL (1988). Comparing the areas under two or more correlated receiver operating characteristic curves: a nonparametric approach. Biometrics.

[B63] Sing T, Sander O, Beerenwinkel N, Lengauer T (2005). ROCR: visualizing classifier performance in R. Bioinformatics.

[B64] Flamm C, Hofacker IL, Maurer-Stroh S, Stadler PF, Zehl M (2001). Design of multistable RNA molecules. RNA.

