# Supplementary material for: Strategies for measuring evolutionary conservation of RNA secondary structures
Source: BMC Bioinformatics. 2008 Feb 26;9:122. doi: 10.1186/1471-2105-9-122 (PMC2335298; doi:10.1186/1471-2105-9-122)
Supplement: Additional file 1 — Overview of the BRAliBase 2.1 dataset. Overview of the BRAliBase 2.1 data set. The number of the alignments in the different entropy bins are shown. The red line indicates the minimal threshold of positive instances we used to obtain reasonable significance levels in the ROC analysis. Bins below this threshold were not considered. [file 1471-2105-9-122-S1.pdf]

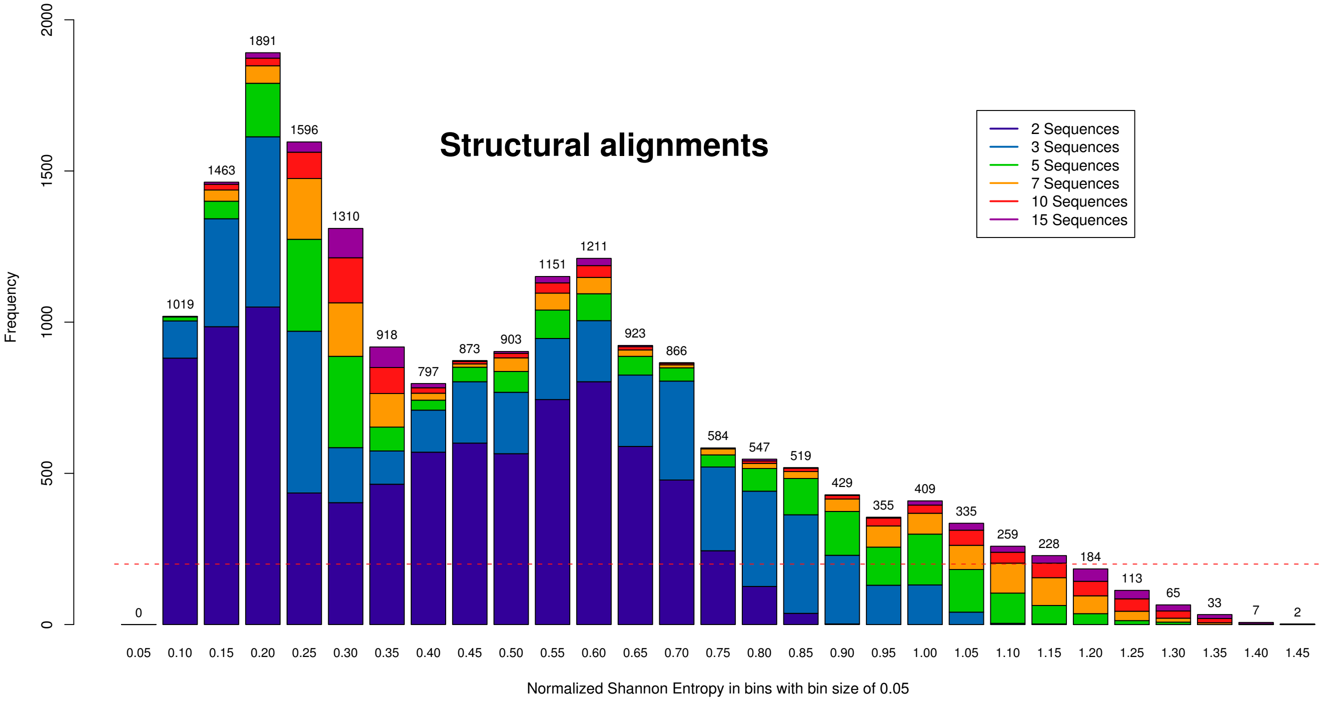

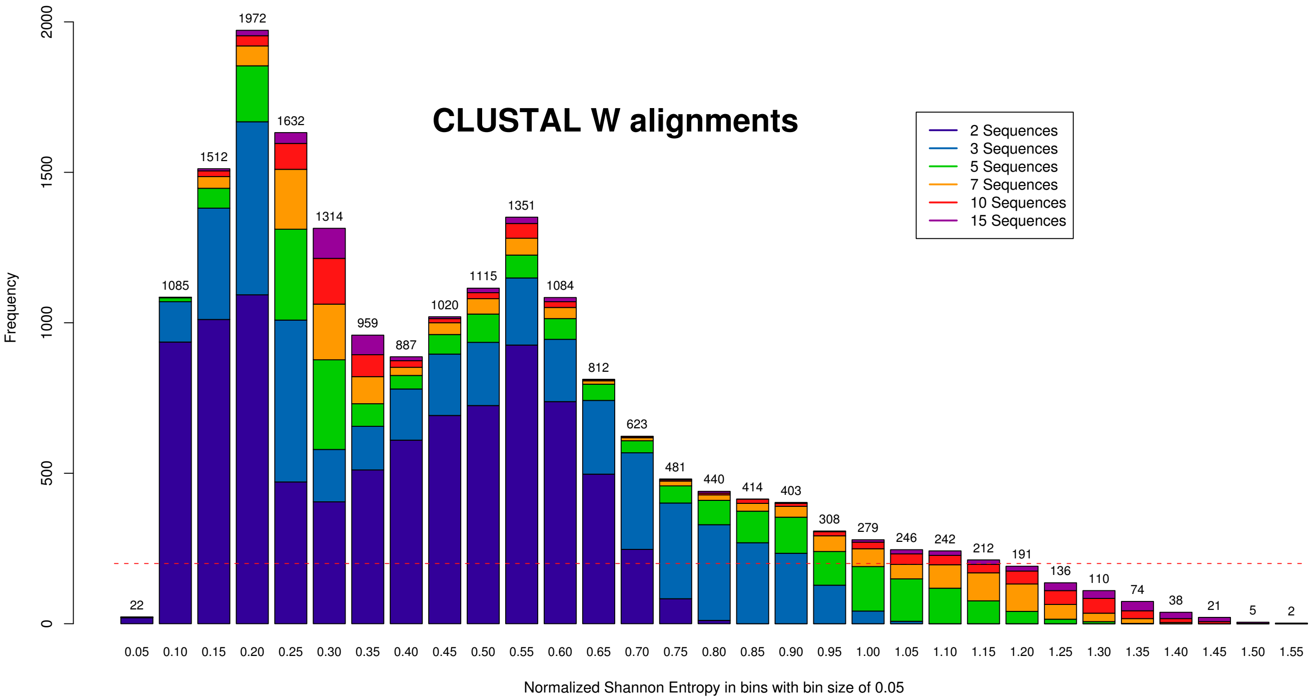

Additional file 1: Overview of the BRAliBase 2.1 data set. The number of the alignments in the different entropy bins are shown. (cf. also Fig. 1). The red line indicates the minimal threshold of positive instances we used to obtain reasonable significance levels in the ROC analysis. Bins below this threshold were not considered.
